# Breastfeeding may have a long-term effect on oral microbiota: results from the Fin-HIT cohort

**DOI:** 10.1186/s13006-020-00285-w

**Published:** 2020-05-15

**Authors:** Ilana Eshriqui, Heli T. Viljakainen, Sandra R. G. Ferreira, Sajan C. Raju, Elisabete Weiderpass, Rejane A. O. Figueiredo

**Affiliations:** 1 Graduation Program in Public Health Nutrition, School of Public Health, University of São Paulo, São Paulo, Brazil; 2 Hospital Israelita Albert Einstein, São Paulo, SP Brazil; 3 Folkhälsan Research Center, Topeliuksenkatu 20, FI-00250 Helsinki, Finland; 4 Department of Food and Nutrition, University of Helsinki, Helsinki, Finland; 5 Department of Epidemiology, School of Public Health, University of São Paulo, São Paulo, Brazil; 6 Faculty of Medicine, University of Helsinki, Helsinki, Finland; 7 International Agency for Research on Cancer, Lyon, France

**Keywords:** Microbiota, Saliva, Breastfeeding, Adolescent

## Abstract

**Background:**

Breastfeeding contributes to gastrointestinal microbiota colonization in early life, but its long-term impact is inconclusive. We aimed to evaluate whether the type of feeding during the first six months of life was associated with oral microbiota in adolescence.

**Methods:**

This is a cross-sectional sub-study using baseline information of 423 adolescents from the Finnish Health in Teens (Fin-HIT) cohort. Type of feeding was recalled by parents and dichotomized as (i) No infant formula; (ii) Infant formula (breastmilk + formula or only formula). Saliva microbiota was analysed using 16S rRNA (V3–V4) sequencing. Alpha diversity and beta diversity were compared between feeding type groups using ANCOVA and PERMANOVA, respectively. Differential bacteria abundance was tested using appropriate general linear models.

**Results:**

Mean age and body mass index were 11.7 years and 18.0 kg/m^2^, respectively. The No formula group contained 41% of the participants. *Firmicutes* (51.0%), *Bacteroidetes* (19.1%), and *Proteobacteria* (16.3%) were the most abundant phyla among all participants. Alpha and beta diversity indices did not differ between the two feeding groups. Three Operational Taxonomic Units (OTUs) belonging to *Eubacteria* and *Veillonella* genera (phylum *Firmicutes*) were more abundant in the No formula than in the Infant formula group (log2fold changes/ *p* - values − 0.920/ < 0.001, − 0.328/ 0.001, − 0.577/ 0.004).

**Conclusion:**

Differences exist in abundances of some OTUs in adolescence according to feeding type during the first six months of life, but our findings do not support diversity and overall oral microbiota composition in adolescents being affected by early feeding type.

## Background

The Developmental Origins of Health and Disease (DOHaD) theory has been a target of many studies to explain the global epidemic of non-communicable diseases. DOHaD theory proposes that exposures during critical development periods, such as the first 1000 days of life, could unleash metabolic programming that is able to modify structure and function of organs and systems, impacting health status later in life [1–3].

Recently, the role of microbiota in the context of DOHaD has been evaluated [4]. Gut microbiota is known to be established during the first two years of life, reaching stability by the third year [5, 6]. Perturbations of microbiota during early life have been associated with later inflammatory or immune-mediated diseases, such as allergy, asthma, and obesity [7–10], indicating that microbiota also has a critical period of development.

Evidence of possible protection of breastfeeding against obesity and related outcomes in adulthood supports the DOHaD theory [11, 12]. An explanation for the protective effect is its role during the gut microbiota establishment period [7, 13]. Relative to formula-fed infants, breastfed infants seem to have a lower abundance of *Clostridium* and a predominance of *Bifidobacteria* and *Lactobacilli* in the gut [6, 8, 14–18].

Studies have suggested that also the infant oral microbiota is influenced by the type of feeding [19–21]. *Proteobacteria* and *Actinobacteria* phyla were more abundant in the oral cheek of breastfed neonates, while formula-fed infants had a higher predominance of *Bacteriodetes* [20]. In examining saliva culture data of three-month-old infants, vital *Lactobacillus* species were found in breastfed but not formula-fed ones [19]. A longer term effect, assessed at four and 12 months after birth, reinforced differences in oral microbiota composition between breast and formula-fed infants [21].

Although differences exist between gut and oral microbiota compositions, some similarities have been described [22–24]. Community types present in these sites seem to be associated, i.e. types of bacteria detected in one site are able to predict those found in the other [22, 23]. It has been suggested that oral microbiota seeds gut microbiota in infancy. Observations that both mother’s milk and infant’s faeces are colonized by some identical bacteria support the hypothesis that human milk is an important source of bacteria, contributing to define gut microbiota composition [22].

Considering that oral microbiota is relatively stable over time in healthy individuals, that perturbations could favour non-oral diseases such as type 2 diabetes [24, 25], and that saliva is easily collected, the use of saliva samples represents a unique opportunity to investigate factors associated with microbial composition. As far as we know, no study has evaluated whether the association between breastfeeding and oral microbiota composition is maintained until adolescence, which is a critical period in the life course for the prevention of chronic diseases. We aimed to evaluate whether the type of feeding during the first six months of life is associated with oral microbiota diversity and composition in Finnish adolescents. Our hypothesis is that those who received infant formula at early life (combined or not with breastmilk) had a lower exposure to breastmilk (in quantitative terms) compared to those who were fed only with breastmilk, which could influence the microbiota development.

## Methods

### Design and study population

This is a cross-sectional sub-study using baseline data from the Finnish Health in Teens (Fin-HIT) cohort [26]. Briefly, Fin-HIT is a representative study of the most populated areas of Finland, for which approximately 11,400 adolescents and 9900 parents (one per adolescent, mostly mothers) were recruited between 2011 and 2014. The Fin-HIT study protocol was approved by the Ethics Committee of the Hospital District of Helsinki and Uusimaa (169/13/03/00/10), and all participants provided informed consent.

**Fig. 1:**
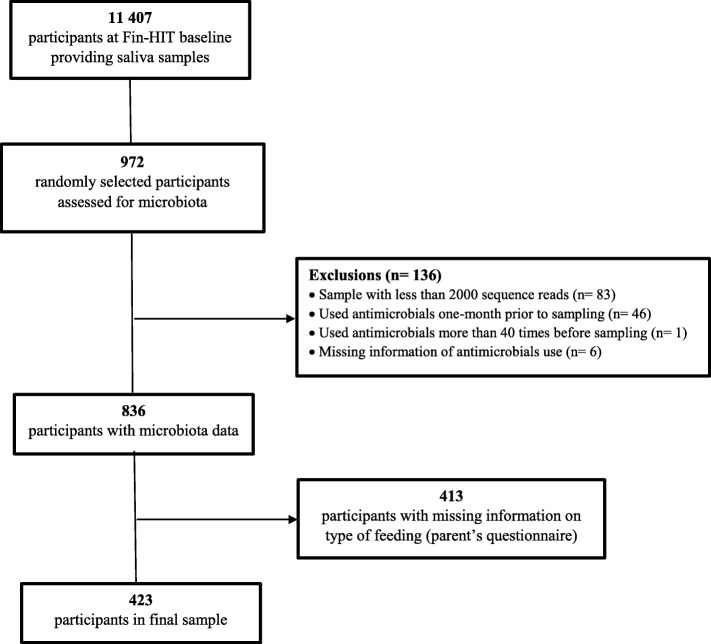
Flow chart of Fin-HIT participants included in this study

Saliva samples from all adolescents were collected and a random subsample (*n* = 972) had the oral microbiota analysed, maintaining representativeness of the cohort population. Participants whose samples generated less than 2000 sequence reads (*n* = 83) were excluded. Based on register data from the Social Insurance Institution of Finland, those who had used antimicrobials in the last month (*n* = 46) or had used them for more than 40 times throughout life (taken as a proxy of chronic disease) (*n* = 1) or without this information were also excluded (*n* = 6). Thus, 836 adolescents had saliva microbiota data, 423 (aged 10–14 years) of whom also had information on feeding type during the first six months of life, comprising the final number of participants in this study (Fig. 1).

### Variables

The type of feeding during the first six months of life was the main exposure of this analysis. At baseline, these data were retrospectively collected using a web questionnaire answered by parents. This variable was dichotomized as follows: (i) No infant formula and (ii) Infant formula (referring to those who received formula, combined or not with breastmilk).

Other variables of interest were adolescent’s gender (male/female) and age (years); and parent’s gender (male/female), age (years), language (Finnish, Swedish/others) and education level (high school or technical level/university degree). Adolescent’s height (nearest 0.1 cm) and weight (nearest 0.01 kg) were measured by trained fieldworkers using portable stadiometers (Seca model 217) and portable digital scales (CAS model PB). Body mass index (BMI) was calculated as weight (kg)/height^2^ (m). Type of delivery (vaginal/ C-section) was obtained from the national health register managed by the National Institute for Health and Welfare [https://www.thl.fi/en/web/thlfi-en/statistics/ information-for-researchers].

### Saliva collection and oral microbiome analysis

Adolescents’ unstimulated saliva samples were collected using the Oragene® DNA (OG-500) Self-Collection Kit (DNA Genotek Inc., Ottawa, ON, Canada), mixed with stabilizing reagent and stored at ambient temperature, according to the manufacturer’s instructions. A protocol with an intensive lysis step and mechanical disruption of microbial cells was employed [27]. Afterwards, total DNA was extracted; sample amplification and sequencing were prepared according to a simplified in-house 16S rRNA gene-based PCR amplification protocol. Amplification was performed using 16S primers targeting V3-V4 region and the Truseq (TS)-tailed1-step amplification protocol. The Illumina HiSeq1500 instrument (Illumina Inc., San Diego, CA, USA) was used for PCR amplicons sequencing. MiSeq SOP in the mothur pipeline (version V.1.35.1) was used to process sequences [28]. The SILVA 16S rRNA database (version V119) and taxonomy were used for alignment and classification of the high-quality sequence reads, which were clustered into operational taxonomic units (OTUs) at a cut-off value > 98% sequence similarity. Detailed procedures of saliva collection and microbial analysis were previously described [29].

Alpha diversity (Shannon and Inverse Simpson Indices) was calculated per sample and beta diversity between the samples using Bray Curtis dissimilarity indices. Sequencing depths were categorized as (i) low ≤10,000; (ii) medium > 10,000 and ≤ 100,000; (iii) high > 100,000 sequences.

### Statistical analysis

Kolmogorov-Smirnov test was used to verify normal distribution of variables, which were described with means (standard deviation) or frequency (%). Student t-test and Chi-squared test were used to compare continuous and categorical variables according to feeding type groups. ANCOVA was used to compare alpha diversity between groups. Pearson correlation was employed to test correlations between alpha diversity indices and continuous variables, while Student t-test and ANOVA were used to compare these indices between categories of the other variables. These analyses were performed using Stata Statistical Software (release 12, 2011, StataCorp LP, College Station, TX, USA), and statistical significance was set at the level of 5 %. Permutational analysis of variance (PERMANOVA) was used to test difference in beta diversity between feeding type groups. Principal coordinate analysis based on Bray Curtis distances was used to illustrate beta diversity between groups.

General linear models (GLMs) with negative binomial distribution were employed for comparisons of bacteria abundance between feeding type groups as OTU and considering phylum and genus levels. All OTUs with low counts (< 20) were excluded. The *P* - values were corrected by false discovery rate. PERMANOVA and GLM were carried out using DESeq2 [30], Vegan, and phyloseq in R (version 3.4.3). Gender, age, BMI, type of delivery, parent’s education, and sequence reads were considered confounders in all adjusted models.

Multiple imputation for missing values of BMI (*n* = 10), type of delivery (*n* = 22), and parent’s education (*n* = 15) was performed with “mi impute chained” procedure in Stata 12.0. Imputed values were considered in the multivariate analysis.

## Results

Of the 423 adolescents at baseline of Fin-HIT, 52% were female; their mean age and BMI were 11.7 years and 18.0 kg/m^2^ (73% were normal weight), respectively. Most participants were born by vaginal delivery (81.1%) and received infant formula (solely or combined with breastmilk) (58.6%) during their first six months of life. The majority of participating parents were female (87.4%), Finnish speakers (90.3%), and had a university degree (59.6%) (Table 1).

**Table 1:** Main data of participants at Fin-HIT baseline and comparison according to type of feeding

	All *n* = 423	No infant formula *n* = 175	Infant formula *n* = 248	***P*** - value
**Continuous variables**	**Mean (SD)**			
Adolescent’s age (y)	11.7 (0.3)	11.7 (0.3)	11.7 (0.3)	0.074
Parent’s age (y)	44.0 (5.7)	44.3 (5.4)	43.8 (5.8)	0.389
Adolescent’s body mass index (kg/m^2^) ^a^	18.0 (2.9)	17.7 (2.8)	18.2 (2.9)	0.077
Alpha diversity ^b^				
Shannon Index	2.9 (0.3)	2.9 (0.3)	2.9 (0.3)	0.877
Inverse Simpson	10.1 (3.1)	10.1 (3.2)	10.1 (2.3)	0.949
**Categorical variables**	**Frequency (%)**			
Adolescent’s gender				
Male	204 (48.2)	82 (46.9)	122 (49.2)	0.636
Female	219 (51.8)	93 (53.1)	126 (50.8)	
Type of delivery ^c^				
Vaginal	325 (81.1)	144 (88.3)	181 (76.1)	**0.002**
C-section	76 (18.9)	19 (11.7)	57 (23.9)	
Parent’s language				
Finnish	382 (90.3)	156 (89.1)	226 (91.1)	0.638
Swedish	36 (8.5)	16 (9.2)	20 (8.1)	
Other	5 (1.2)	3 (1.7)	2 (0.8)	
Parent’s education ^d^				
High school/ technical level	165 (40.4)	72 (43.4)	93 (38.4)	0.318
University degree	243 (59.6)	94 (56.6)	149 (61.6)	

^a^*n* = 413 due to missing data

^b^ Adjusted for adolescent’s gender, age, and body mass index, type of delivery, parent’s education, and sequence reads

^c^*n* = 401 due to missing data

^d^*n* = 408 due to missing data

**Fig. 2:**
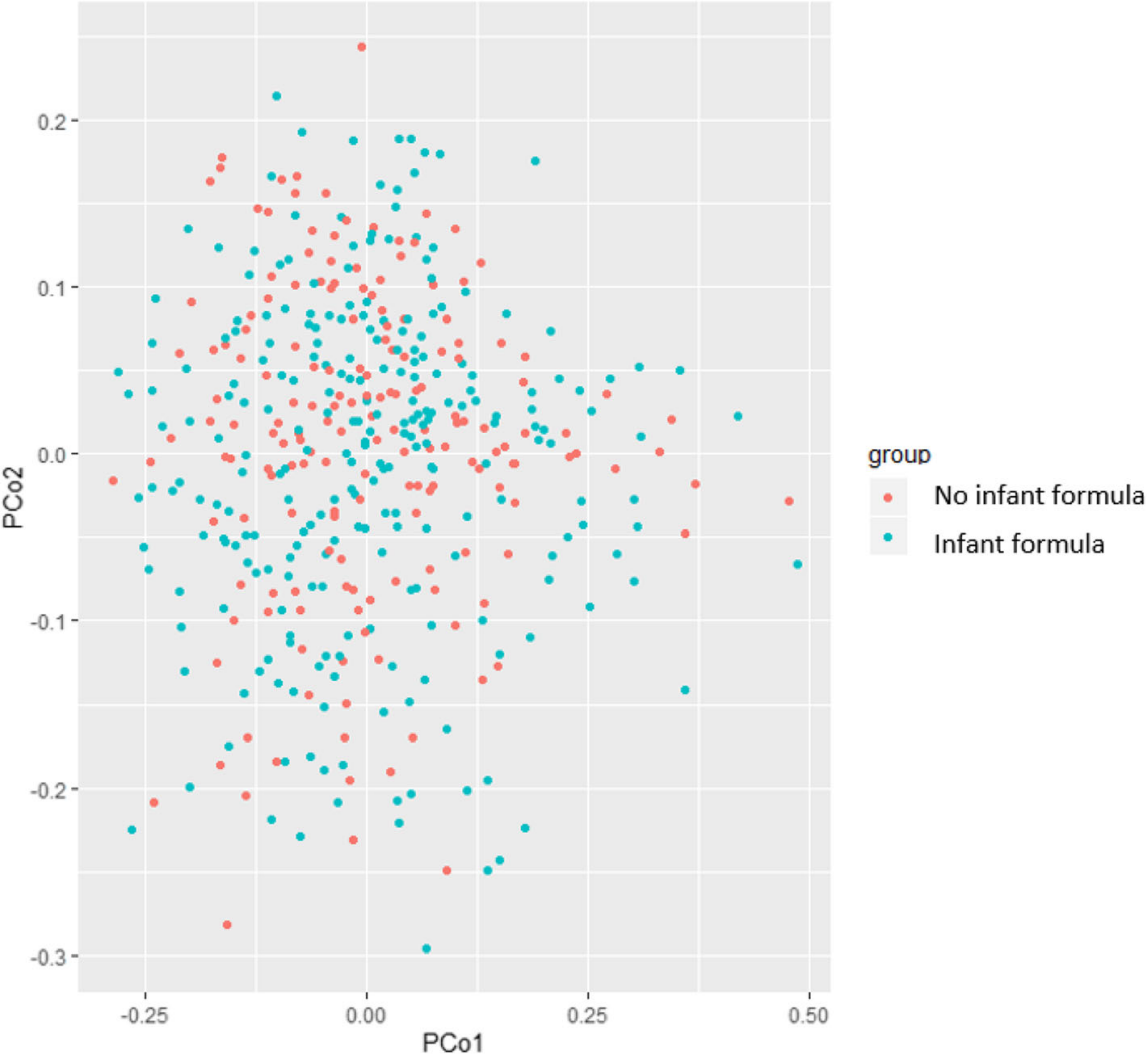
Principal coordinate analysis (beta-diversity) for the saliva microbiota according to type of feeding (*P –* value = 0.881)

Mean (standard deviation) values of alpha diversity indices were 2.9 (0.3) and 10.1 (3.1) for Shannon and Inverse Simpson, respectively, and these did not differ between the No formula and Infant formula groups (Table 1). Beta diversity was also similar between groups (*P* - value = 0.881) (Fig. 2). In the No formula group, vaginal delivery was more frequent than in the Formula group (88.3% vs. 76.1%, *P* - value = 0.002) (Table 1). Alpha diversity was not associated with adolescents’ or parents’ characteristics (Table 2).

**Table 2:** Correlations and comparison of mean values of diversity indices by participants’ characteristics

	Shannon		Inverse Simpson	
**Continuous variables**	**r**	***P*- value**	**r**	***P*- value**
Adolescent’s age	0.015	0.763	−0.003	0.949
Adolescent’s body mass index	−0.047	0.344	−0.037	0.456
**Categorical variables**	**Mean (SD)**	***P*- value**	**Mean (SD)**	***P*- value**
Adolescent’s gender				
Male	2.9 (0.3)	0.338	10.2 (3.0)	0.412
Female	2.9 (0.3)		9.9 (3.2)	
Type of delivery				
Vaginal	2.9 (0.3)	0.579	10.1 (3.1)	0.579
C-section	2.9 (0.3)		9.9 (3.0)	
Parent’s language				
Finnish	2.9 (0.3)	0.776	10.0 (3.1)	0.802
Swedish	2.9 (0.2)		10.2 (3.0)	
Other	3.0 (0.4)		10.9 (4.1)	
Parent’s education				
High school/ technical level	2.9 (0.3)	0.544	9.8 (3.0)	0.190
University degree	2.9 (0.3)		10.2 (3.2)	
Sequence reads				
Low	2.8 (0.2)		9.3 (2.1)	
Medium	2.9 (0.3)	0.274	10.0 (2.9)	0.360
High	2.9 (0.4)		10.3 (3.4)	

**Fig. 3:**
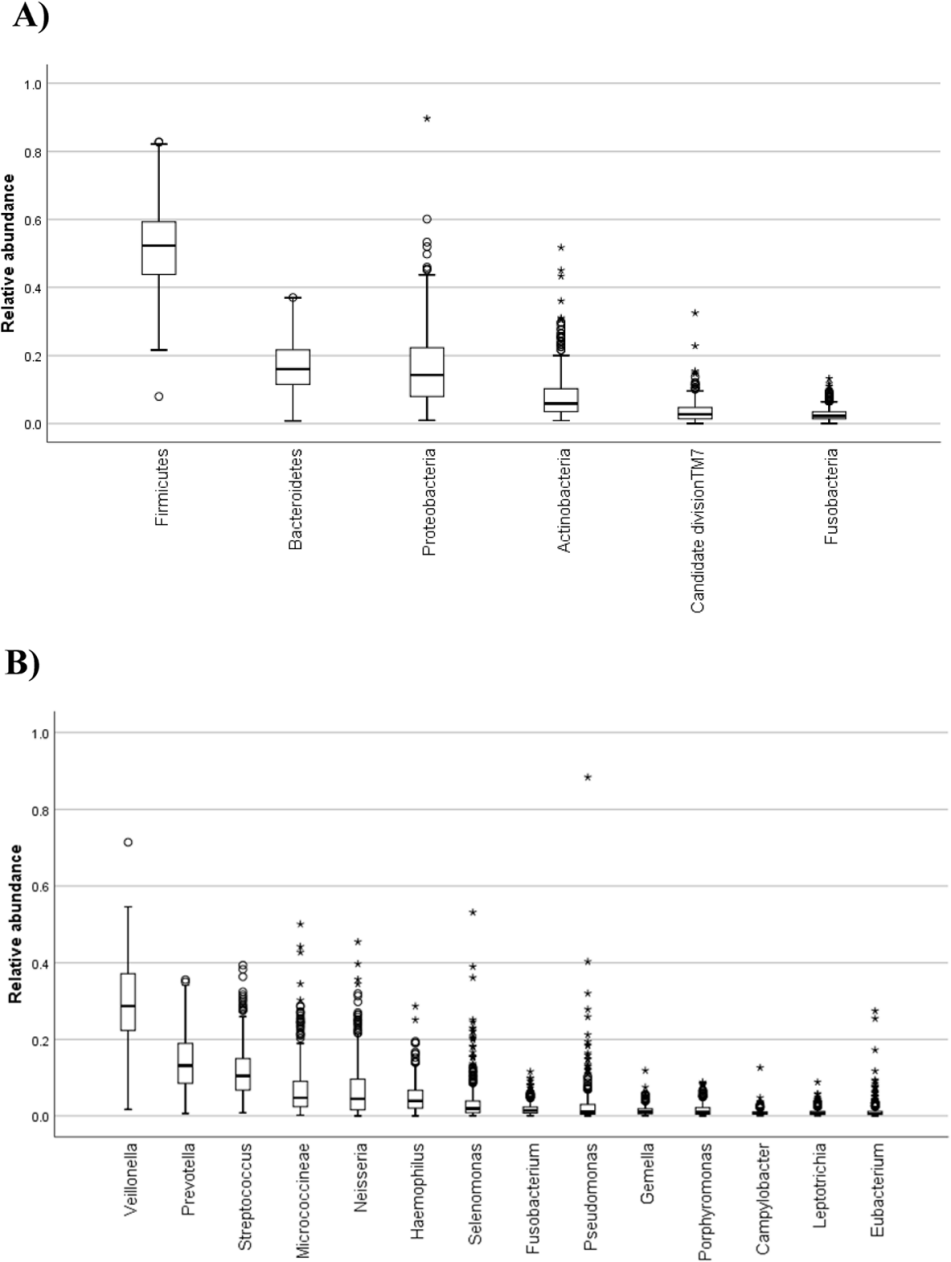
Relative abundances of phyla (**a**) and genera (**b**) in saliva microbiota

In 423 samples of adolescents’ saliva, 22,924,455 raw sequences reads were obtained and 1049 OTUs were evaluated. In total, 11 phyla, 16 classes, 24 orders, 44 families, and 76 genera were identified. As depicted in Fig. 3, a great predominance of phylum *Firmicutes* was found, followed by *Bacteroidetes*, *Proteobacteria,* and *Actinobacteria* (panel A), while the predominant genera were *Veillonella, Prevotella,* and *Streptococcus* (panel B).

**Fig. 4:**
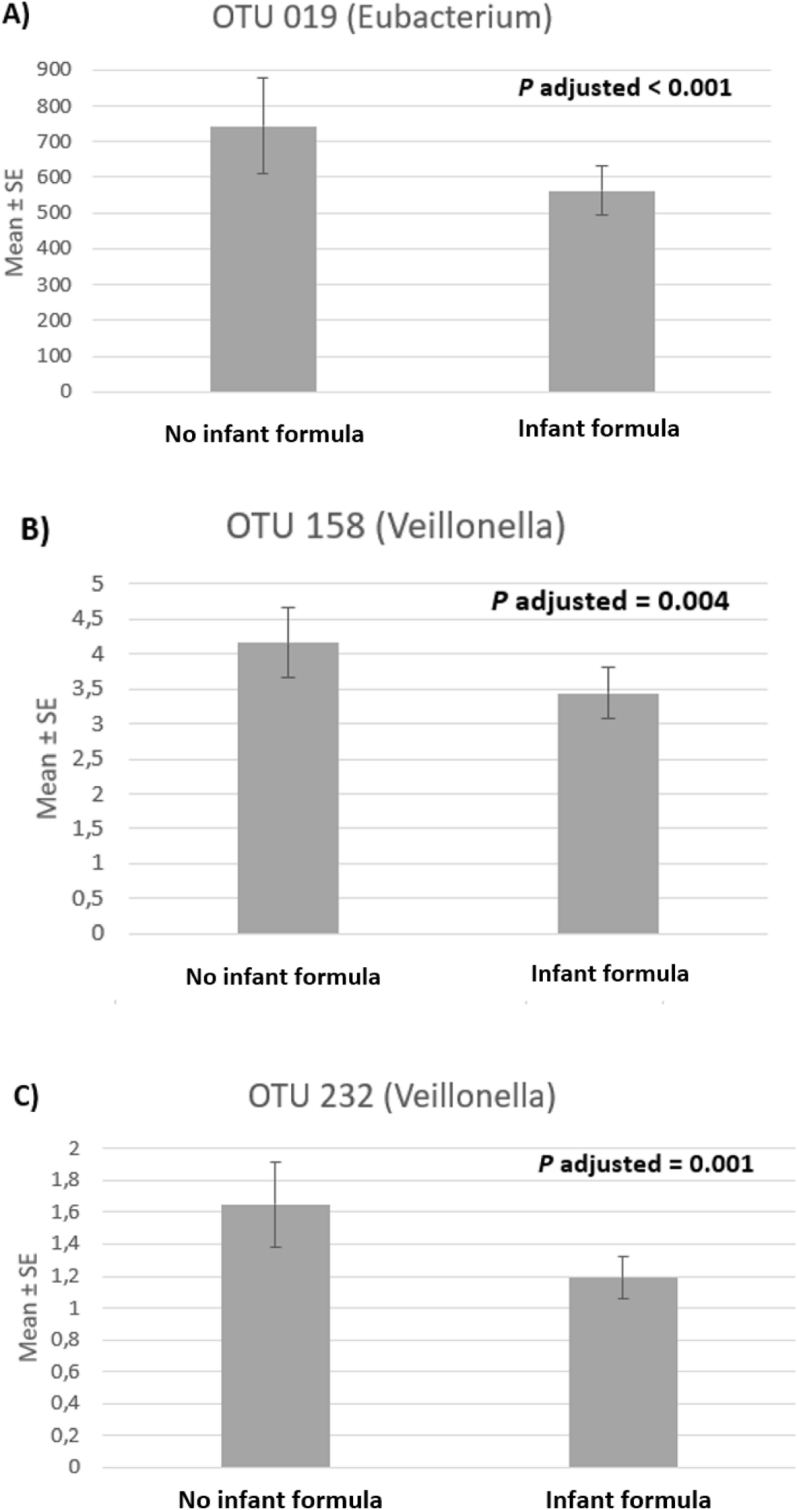
Means and standard errors (SEs) of abundance for significantly different OTUs between feeding type groups

Comparing groups of feeding type according to abundance of bacteria, no difference was observed at phylum level. Three OTUs (#019, #232, and #158) were more abundant in No formula than in Infant formula group (log2fold changes/ adjusted *p*-values: − 0.920/ < 0.001, − 0.328/ 0.001, and − 0.577/ 0.004, respectively) (Fig. 4; and see table at Additional file 1 for detailed information). These three OTUs belonged to phylum Firmicutes, class *Clostridia,* and order *Clostridiales*; OTU #019 belonged to genus *Eubacteria* and #232 and #158 to genus *Veillonella*. Considering the entire genera, genus *Eubacteria* (log2fold change − 0.742, adjusted *p* - value < 0.0003), but not genus *Veillonella*, was more abundant in the No formula group.

## Discussion

We investigated the long-term association of early life feeding type with oral microbiota composition in adolescence. A few differences emerged in abundances of some specific genera according to early feeding type, but our data do not suggest a significant impact on the diversity of microbiota. Abundances of commensal OTUs belonging to genera *Veillonella* and *Eubacteria* were higher in adolescents who had not been fed with formula than in others who had received infant formula, combined or not with breastmilk.

To the best of our knowledge this is the first study to explore associations of early feeding type with oral microbiota diversity and composition at this stage of life. Such relationships have been investigated mainly during the first year of life [19–21]. Despite higher abundance of certain OTUs, the overall composition (beta diversity) or the alpha diversity of oral microbiota did not differ between adolescents with different early life feeding type. In general, high phylogenetic diversity in gut microbiota has been associated with several health outcomes [31], but long-term health implications of this picture are unclear yet. We speculate that our negative results regarding overall microbiota diversity and composition could result from a stronger impact of other exposures during childhood such as weaning age, time of introduction of solid foods, Westernized eating pattern and use of antimicrobials [18, 31–33].

As far as the gut is concerned, it was reported that microbiota composition in breastfed babies tended to be more stable and has less diverse bacterial community compared with formula-fed babies [34, 35]. From three years of age, their composition converge to resemble that of an adult gut [5]. A multicentre cohort of 903 infants (including Finns) followed from three to 46 months of age showed that breastfeeding explained the greatest part of microbiome establishment during the first year of life. Also, breastfed infants had lower diversity than those weaned at an earlier stage. As age increased and breastmilk exposure decreased, diversity became more similar between groups, supporting the hypothesis that weaning is an important event affecting maturation of the gut microbiome [6]. Some studies have evaluated the long-term effect of breastfeeding on gut microbiota [6, 18]. A prospective birth cohort study of 281 Dutch children reported that breastfeeding duration was associated with gut microbiota composition in children aged 6–9 years [18].

Little is known how these changes occur in saliva, driven by diet, physical activity and antibiotics administration, which could alter the host health status. Although gut and oral microbiota had distinct compositions, community types of these sites seem to be associated [22, 24]. A study conducted with neonates 20 days old, found a positive correlation of subdominant family *Lactobacillaceae* in saliva and faeces samples of the same infant, and showed that *Staphylococcus spp*. and *Streptococcus spp*. were shared between the two ecosystems and also with human milk [22]. Comparable results have been reported in studies assessing gut and oral microbiota; i.e. early feeding seems to impact on microbiota profile [6, 14, 19, 20].

Comparisons with our findings are limited because we were the first to evaluate the long-term effect of early feeding on oral microbiota. Furthermore, exposures occurring during infancy, particularly related to antibiotics use, may affect microbiota composition mainly at the gut level [36, 37]. The protective role of breastfeeding against increased body adiposity and antibiotic use in childhood, especially by promoting beneficial microbiota, was eliminated by the use of antibiotics in early life [38]. Additionally, differences in microbiota composition from one population to another are recognized [39–41].

According to unpublished data of the Fin-HIT, most of the participants used antimicrobial drugs during their first years of life, which could contribute to a lasting impact on oral microbiota diversity and composition at adolescence. Regarding diet, it is suggested that especially the high and frequent intake of sucrose and other fermentable carbohydrates result in the accumulation of acidogenic and aciduric microorganisms, driving a pathogenic biofilm community formation. Particularly, high frequency of sugar intake could disrupt the homeostasis between commensals and pathogens resulting in dysbiosis, which could increase chances of caries, inflammation and periodontitis in more susceptible individuals [42]. Thus, we suppose that sugar intake during childhood could be an important factor affecting diversity and composition of oral microbiota. A previous study, conducted in public child health service in Finland, showed that sugar introduction occurs early in life. Almost half of six month old children were receiving sugar-sweetened beverages and more than 90% of those older than 16 months were receiving sweets [43]. Further, another Finnish study showed that 95% of one year-old babies consume mass-produced baby foods, of which some are sweetened with juice-concentrates (70–80% of sugar) [44].

Our finding of a major predominance of phylum *Firmicutes* in the human oral cavity, followed by *Bacteroidetes*, *Proteobacteria,* and *Actinobacteria* is consistent with previous studies in one to two month-old [20] and 12 month-old children [21]. A study conducted in 38 healthy, full-term, vaginally delivered neonates from Australia found similar alpha diversity and proportion of *Firmicutes* between breastfed and formula-fed groups (96.3 vs. 95.3%) [20], in accord with our findings in an older age group. A higher relative abundance of *Bacteroidetes* and a lower abundance of *Actinobacteria* and *Proteobacteria* were also observed in formula-fed infants than in breastfed infant.

Holgerson et al. [19] showed that three month old exclusively breastfed infants clustered separately from formula-fed infants, according to their oral microbiota. In total, 14 probes differed significantly between these two feeding types. Among these probes, cultivable *Lactobacilli* and *Eubacterium yurii* were more abundant in breastfed than exclusively formula-fed infants, in whom the mean number of species per child was higher. *Lactobacillus,* but not *Eubacterium,* has been consistently reported in breastmilk, and thus, the presence of the former in infants’ oral microflora was expected. We do not know whether the higher abundance of *Eubacteria* observed in our breastfed adolescents could be detected earlier, during their infancy. Another study reported higher species richness in 4-month-old formula-fed infants and marked differences in saliva microbiota composition between feeding type subgroups [21]. However, at 12 months of age these differences were no longer significant.

We found that *Eubacteria* and *Veillonella*, both genera of the normal oral microbiota, were more abundant in adolescents of No formula group compared with those who received infant formula in early life. *Eubacteria* is an atypical genus in samples of breastmilk, but *Veillonella*, *Gemella, Rothia*, *Lactobacillus*, *Streptococcus, and Staphylococcus* have been previously described as the most abundant genera in breastmilk [45]. The association of No formula with higher *Veillonella* abundance seems favourable, considering that this genus has a beneficial effect on dental plaques by metabolizing lactate to weaker acids such as acetate and propionate [46]. Since lactate is a cariogenic factor produced by *Streptococcus mutans*, increased abundance of *Veillonella* could be interpreted as a compensatory effect to protect against dental injuries. Higher abundance of the species *Veillonella parvula* was previously described in subgingival biofilm samples from healthy subjects compared with those with chronic periodontitis, and its presence was inversely correlated with inflammatory biomarkers from gingival crevicular fluid [47]. Periodontal disease is considered a risk factor for cardiovascular disease, and the proposed underlying mechanism was based on increased circulating pro-inflammatory cytokines [48].

Strict anaerobic bacteria from the genus *Eubacteria* are chemoheterotrophs, i.e. are unable to synthesize their own organic molecules, requiring mixed organic acids from the host. Thus, *Eubacteria* energy is obtained from carbohydrates or protein metabolism, resulting in end-products such as butyrate and acetate [49]. These short-chain fatty acids have been associated with beneficial effects, such as cardiovascular and colonic disease prevention [50]. The presence of Eubacteria in our sample of healthy adolescents was expected since this a member of normal saliva microflora and plaques. Our methods were unable to identify species of *Eubacteria* that would be desirable since periodontal pathogenic *Eubacteria* species were previously described [47, 51]. A study of patients undergoing haemodialysis identified higher taxa of *Eubacterium nucleatum* in sulcular fluid from the periodontal pocket of non-diabetics than diabetics. Despite being a pathogen, the prevalence of periodontitis was similar between the groups [51].

Although evidence has suggested beneficial roles of *Veillonella* and *Eubacteria*, it was not possible to extrapolate these in our sample since the study design does not allow comparing health outcomes according to abundances of specific bacteria. Prospective studies are needed to investigate whether individuals with distinct abundances of specific bacteria have different chances of developing cardiovascular disease or other outcomes.

A strength of this study was the large sample size in which next-generation sequencing technology was used to analyse microbiota. This is a sub-study of a well-designed prospective cohort, including reliable information obtained from standardized questionnaires and national health registers [26]. Statistical analyses were duly adjusted, minimizing confounding bias. The main limitations of our study comprised a design impeding establishment of causality and retrospective data collection (feeding type answered by parents), which could generate recall bias. Considering that we evaluated associations of an event occurred long time ago (type of feeding) and another occurring in adolescence, there is a possibility of recall error, especially among parents who had multiple children. However, it has been reported that information about breastfeeding recalled by mothers after 20 years still shows reasonable accuracy [52]. We could not consider “exclusive breastfeeding” since no information regarding water, tea, and food intake in early life was available, or “exclusive formula-fed” (not combined with breastmilk) since its prevalence in our study was relatively low (2.4%). Previously, these categories of “exclusive” feeding type were clearly separated by microbiota composition, but partially breastfed infants were interspersed between these groups [19]. Despite this limitation that could have attenuated differences, we still detected some differences according to feeding type. We speculate that differences identified are explained by a higher exposure to breastmilk at early life by participants of the No formula group compared to those of the Infant formula group.

Finally, the lack of oral health status could be regarded as a limitation. It is known that diet, especially in combination with poor oral health, salivary dysfunction, scarce fluoride exposure and poor oral hygiene, can modulate oral microbiota resulting in dysbiosis [42]. Since Finland public oral health care services are freely available for individuals younger than 18 years, most children and adolescents have relatively good oral health, although differences exist depending on maternal educational level and region. Not having information on oral health could contribute to confounding bias and thus is a limitation in this study.

## Conclusion

The type of feeding in early life is not associated with overall changes in composition or diversity but with some alterations in adolescents’ oral microbiota (higher abundance of *Veillonella* and *Eubacteria*). The clinical relevance of these findings and their health implications need to explored in future studies. Other exposures during childhood (e.g. e.g. antimicrobial use, sugar consumption and oral health) could play a stronger role in adolescents’ oral microbiota diversity and composition than the early feeding. Further prospective studies, considering the type of feeding in early life, age of weaning, and other factors during childhood, are needed to confirm our findings.

## Supplementary information

**Additional file 1.** Differentially abundant bacteria at OTU-level by type of feeding (No infant formula vs. Infant formula) during the first six months of life.

## Data Availability

The data that support the findings of this study are available from Heli T. Viljakainen but restrictions apply to the availability of these data, which were used under license for the current study, and so are not publicly available. Data are however available from the authors upon reasonable request and with permission of Heli T. Viljakainen.

## Abbreviations

DOHaD: Developmental Origins of Health and Disease
Fin-HIT: Finnish Health in Teens cohort
BMI: Body Mass Index
OTU: Operational Taxonomic Unit
GLM: General Linear Model
