# Supplementary material for: Breastfeeding may have a long-term effect on oral microbiota: results from the Fin-HIT cohort
Source: Int Breastfeed J. 2020 May 15;15:42. doi: 10.1186/s13006-020-00285-w (PMC7227309; doi:10.1186/s13006-020-00285-w)
Supplement: Supplementary file 1 — Additional file 1. Differentially abundant bacteria at OTU-level by type of feeding (No infant formula vs. Infant formula) during the first six months of life. [file 13006_2020_285_MOESM1_ESM.docx]

**Additional file 1.** Differentially abundant bacteria at OTU-level by type of feeding (No infant formula vs. Infant formula) during the first six months of life.

| **OTU (nearest taxa)** | **Base Mean** | **log2FoldChange** ^a^ | ***P* adjusted** |
| --- | --- | --- | --- |
| OTU 019 (Eubacterium) | 517.592 | -0.920 | 4.49E-05 |
| OTU 158 (Veillonella) | 2.820 | -0.328 | 0.004 |
| OTU 232 (Veillonella) | 1.103 | -0.577 | 0.001 |

^a^ Negative value means lower abundance among “Infant formula” group compared to “No infant formula”.
